# Supplementary material for: A Critical View of the Application of the APEX Software (Aqueous Photochemistry of Environmentally-Occurring Xenobiotics) to Predict Photoreaction Kinetics in Surface Freshwaters
Source: Molecules. 2019 Dec 18;25(1):9. doi: 10.3390/molecules25010009 (PMC7017383; doi:10.3390/molecules25010009)
Supplement: Supplementary file 1 [file molecules-25-00009-s001.zip › 3.2.4_gcc-4.4.0.zip!/3.2.4_gcc-4.4.0/3.2.4_gcc-4.4.0/share/octave/packages/io-1.0.11/doc/READ-XLS.html]

README
for Excel spreadsheet file r/w access scripts for octave (>
3.2.0)  
  
Copyright (C) 2009, 2010 Philip Nienhuis <prnienhuis
at users.sf.net>  
  
This version 1 March 2010.

**EXCEL
.XLS SUPPORT FILES**

**doc/README-XLS.html**

This
file.

**xlsread.m**

All-in-one
function for reading data from one specific worksheet in an Excel
spreadsheet file. This script has Matlab-compatible
functionality.  
  
**xlswrite.m**

All-in-one
function for writing data to one specific worksheet in an Excel
spreadsheet file. This script has Matlab-compatible functionality.
  
  
**xlsfinfo.m**

All-in-one
function for exploring basic properties of an Excel spreadsheet file.
This script has Matlab-compatible functionality.  
  
**xlsopen.m**

Function
for "opening" (= providing a handle to) an Excel
spreadsheet file ("workbook"). This function sorts out
which interface to use for .xls access (i.e.,COM; Java & Apache
POI; JexcelAPI; etc.), but it's choice can be overridden.  
  
**xls2oct.m**

Function
for reading data from a specific worksheet pointed to in a struct
created by xlsopen.m. xls2oct can be called multiple times
consecutively using the same pointer struct, each time allowing to
read data from different ranges and/or worksheets. Data are returned
in the form of a 2D heterogeneous cell array that can be parsed by
parsecell.m. xls2oct is a mere wrapper for interface-dependent
scripts that do the actual low-level reading .  
  
**oct2xls.m**

Function
for writing data to a specific worksheet pointed to in a struct
created by xlsopen.m. octxls can be called multiple times
consecutively using the same pointer struct, each time allowing to
write data to different ranges and/or worksheets. oct2xls is a mere
wrapper for interface-dependent scripts that do the actual low-level
writing.  
  
**xlsclose.m**

Function
for closing (the handle to) an Excel workbook. When data have been
written to the workbook oct2xls will write the workbook to disk.
Otherwise, the file pointer is simply closed and possibly used
interfaces for Excel access (COM/ActiveX/Excel.exe) will be shut down
properly.  
  
**parsecell.m**

Function
for separating the data in raw arrays returned by xls2oct, into
numerical/logical and text (cell) arrays.

**REQUIRED
SUPPORT SOFTWARE**

For
the Excel/COM interface:

- A
  windows computer with Excel installed

  - Octave-forge
    Windows-1.0.8 package WITH LATEST SVN PATCHES APPLIED

For
the Java / Apache POI / JExcelAPI interfaces (general):

- octave-forge
  java-1.2.5 package or later version on Linux

  - octave-forge
    java-1.2.6 with latest svn fixes on Windows/MingW

    - Java
      jre or jdk > 1.6.0 (hasn't been tested with earlier versions)

Apache
POI specific:

- class
  .jars: **poi-3.5-FINAL-<date>.jar** &
  **poi-ooxml-3.5-FINAL-<date>.jar** (or later versions) in
  classpath

  - Get
    it here: http://poi.apache.org/download.html

    - for
      OOXML support (only available with Apache POI):

      poi-ooxml-schemas-<version>.jar,
      xbean.jar, dom4j-1.6.1.jar in javaclasspath.

      Get
      them here:

      http://poi.apache.org/download.html
      (“xmlbeans” and poi-ooxml-schemas)

      http://sourceforge.net/projects/dom4j/files
      (dom4j-<version>)

JExcelAPI
specific:

- class
  .jar: **jxl.jar** in classpath

  - Get
    it here: http://sourceforge.net/projects/jexcelapi/files/

Hint:
simply put the relevant javaaddpath statements in the .octaverc file.

**USAGE**

**xlsread**
and **xlswrite** are mere wrappers for
**xlsopen**-**xls2oct**-**xlsclose**-**parsecell** and
**xlsopen**-**oct2xls**-**xlsclose** sequences, resp. They
exist for the sake of Matlab compatibility.  
  
**xlsfinf**o
can be used for finding out what worksheet names exist in the file.
For OOXML files the input parameter REQINTF should be specified with
a value of 'poi' (case-insensitive) and -obviously- the complete POI
interface must have been installed.  
  
Invoking
**xlsopen**/..../**xlsclose** directly provides for much more
flexibility, speed, and robustness than **xlsread** / **xlswrite**.
Indeed, using the same file handle (pointer struct) you can mix
reading & writing before writing the workbook out to disk using
xlsclose.  
And: **xlsopen** / **xlsclose** hide the gory
interface details from the user.  
Currently only .xls files (BIFF8)
can be read/written; using JExcelAPI BIFF5 can be read as well. For
OOXML files the complete Apache POI interface must be installed (and
possibly the REQINTF parameter specified with a value of
'poi').  
  
When using **xlsopen**....**xlsclose** be sure
to keep track of the file handle struct.  
  
A possible
scenario:  
  
**xlh = xlsopen (<excel\_filename> , [rw],
[<requested interface>])**  
*# Set rw to 1 if you want to
write to a workbook immediately.  
# In that case the check for file
existence is skipped and  
# -if needed- a new workbook created.  
#
If you really want an other interface than auto-selected  
# by
xlsopen you can request that. But xlsopen still checks  
# proper
support for your choice.*  
  
*# Read some data*  
**[
rawarr1, xlh ] = xls2oct (xlh, <SomeWorksheet>, <Range>)**  
*#
Be sure to specify xlh as output argument as xls2oct keeps  
# track
of changes and the need to write the workbook to disk   
# in the
xlhstruct. And the origin range is conveyed through  
# the xlh
pointer struct.*  
  
*# Separate data into numeric and text
data*  
**[ numarr1, txtarr1, lim1 ] = parsecell (rawarr1)**  
#
Get more data from another worksheet in the same workbook  
**[
rawarr2, xlh ] = xls2oct (xlh, <SomeOtherWorksheet>, <Range>)**  
**[
numarr2, txtarr2, lim2 ] = parsecell (rawarr2)**  
  
# <...
Analysis and preparation of new data in cell array Newdata....>  
  
*#
Add new data to spreadsheet*  
**xlh = oct2xls (Newdata, xlh,
<AnotherWorksheet>, <Range>)**  
  
*# Close the
workbook and write it to disk; then clear the handle*  
**xlh =
xlsclose (xlh)**  
**clear xlh**

When
not using the COM interface, specify a value of 'POI' for parameter
REQINTF when accessing OOXML files in xlsread, xlswrite, xlsopen,
xlsfinfo (and be sure the complete Apache POI interface is
installed).

**MATLAB
COMPATIBILITY**

**xlsread**,
**xlswrite** and **xlsfinfo** are for the most part
Matlab-compatible. Some small differences are mentioned
below.  
  
**xlsread**  
Matlab's **xlsread** supports
invoking extra functions while reading ("passing function
handle"); octave not. But this can be simulated outside
**xlsread**.  
  
Matlab's **xlsread** flags some spreadsheet
errors, octave-forge just returns blank cells.  
  
When using a
Java interface, octave-forge returns info about the actual (rather
than the requested) cell range where the data came from; Matlab and
ActiveX/Excel can't. This is due to Excel automatically trimming
returned arrays from empty outer columns and rows. When using COM,
octave has the same limitation.  
Personally I find it very useful
to know from what part of a worksheet the data originate so I've put
quite some effort in it :-)

Matlab's
xlsread ignores all non-numeric data values outside the smallest
rectangle encompassing all numerical values. Octave's xlsread
doesn't. This means that Matlab ignores all row/column headers, not
very user-friendly IMO.   
  
When using the Java interface,
reading and writing xls-files by octave-forge is
platform-independent. On systems w/o Excel Matlab can only read Excel
95 formatted .xls files.  
  
Matlab's **xlsread** invokes
**csvread** if no Excel interface is present. Octave-forge's
**xlsread** doesn't.  
  
**xlswrite**  
Octave-forge's
**xlswrite** works on systems w/o Excel support, Matlab's doesn't
(properly).

When
specifying a sheet number larger than the number of existing sheets
in an .xls file, Matlab's xlswrite adds empty sheets until the new
sheet number is created; Octave's xlswrite only adds one sheet called
“Sheet<number>” where <number> is the
specified sheet number.

Even
better (IMO) while M's xlswrite always creates Sheet1/Sheet2/Sheet3
when creating a new spreadsheet, octave's xlswrite only creates the
requested worksheet.

And
octave doesn't touch the “active sheet”.

If
the specified write range is larger than the actual data array,
Matlab's xlswrite adds #N/A cells to fill up the lowermost rows and
rightmost columns; octave-forge's xlswrite doesn't.   
  
**xlsfinfo**  
When
invoking Excel/COM interface, octave-forge's **xlsfinfo** also
echoes the type of sheet (worksheet, chart), not just the sheet
names. Using Java I haven't found similar functionality (yet).

**COMPARISON
OF INTERFACES & USAGE**

Using
Excel itself (through COM / ActiveX on Windows systems) is probably
the most robust and versatile and especially FAST option. There's one
gotcha: in case of some type of COM errors Excel will keep running
invisibly, you can only end it through Task Manager.  
A tiny
problem is that one cannot find out easily through COM what file
types are supported; xls, wks, wk1, xlsx, etc.  
Another -obvious-
limitation is that COM Excel access only works on Windows systems
where Excel is installed.  
As Excel/COM trims returned arrays from
empty outer row and columns it is not easy to find out from what cell
range the returned data originate.  
  
JExcelAPI (Java-based and
therefore platform-independent) is proven technology but switching
between reading and writing is quite involved and memory-hungry when
processing large spreadsheets. As the docs state, JExcelAPI is
optimized for reading and it does do that well - but slower than
Excel/COM. The fact that upon a switch from reading to writing the
existing spreadsheet is overwritten in place by a blank one and that
you can only get the contents back wen writing out all of the changes
is worrying - and any change after the first write() is lost as a
next write() doesn't seem to work. The first is by JExcelAPI design,
the second is probably a bug (in octave-forge/Java or JExcelAPI ? I
don't know). Adding data to existing spreadsheets does work, but IMO
undue user confidence is needed.

JExcelAPI
supports BIFF5 and BIFF8 (Excel 95 and Excel 97-2003, respectively).

JexcelAPI,
unlike ApachePOI, doesn't evaluate functions while reading but
instead relies on cached results (i.e. results computed by Excel
itself). Depending on Excel settings (“Automatic calculation”
ON or OFF) this may or may not yield incorrect results.  
  
Apache
POI (Java-based and platform-independent too) is based on the
OpenOffice.org I/O Excel r/w routines. It is a little more versatile
than JExcelAPI, while it doesn't support BIFF5 it does support BIFF8
(Excel 97 – 2003) and OOXML (Excel 2007).

It
is slower than native JXL let alone Excel & COM but it features
active formula evaluation, although at the moment (v. 3.6) not all
Excel functions have been implemented. I've made the relevant script
(xls2jpoi2oct) fall back on cached formula results (and yield a
suitable warning) for non-implemented Excel functions while reading
Excel files.  
  
All in all, of the two Java options I'd prefer
Apache POI rather than JexcelAPI. But the latter is indispensable for
BIFF5 formats.

**A
NOTE ON JAVA MEMORY USAGE**

**Java
memory pool allocation size**

The
java virtual machine (JVM) initializes one big chunk of your
computer's RAM in which all java classes and methods etc. are to be
loaded: the java memory pool. It does this because java has a very
sophisticated “garbage collection” system. At least on
Windows, the initial size is 2MB and the maximum size is 64MB. On
Linux this allocated size is much bigger. This part of memory is
where the java-based ODS octave routines (and the java-based xls
routines) live and keep their variables etc.

For
transferring large pieces of information to and from spreadsheets you
might hit the limits of this pool. E.g. to be able to handle I/O of
an array of around 50,000 cells I needed a memory pool size of 512
MB.

The
memory size can be increased by inserting a file called “java.opts”
(without quotes) in the directory
./share/octave/packages/java-<version> (where the script file
javaclasspath.m is located), containing just the following lines:

**-Xms16m**

**-Xmx512m**

(where
16 = initial size, 512 = maximum size, m stands for Megabyte).

After
processing a large chunk of spreadsheet information you might notice
that octave's memory footprint does not shrink so it looks like
java's memory pool does not shrink back; but rest assured, the memory
footprint is the *allocated* (reserved)
memory size, not the actual used size. After the JVM has done its
garbage collection, only the so-called “working set” of
the memory allocation is really in use and that is a trimmed-down
part of the memory allocation pool. On Windows systems it often
suffices to minimize the octave terminal for a few seconds to get a
more reasonable memory footprint.

**DEVELOPMENT**

**xlsopen**/**xlsclos**e
and friends have been written so that adding other interfaces (Perl?
native octave? ...?) should be very easily accomplished. **xlsopen**
merely needs two stanzas, **xls2oct**, **oct2xls** and **xlsfinfo**
each need an additional elseif clause, and **xlsclose** needs a
small stanza for closing the pointer struct and writing to disk.

The
real work lies in creating the relevant **xls2<...>2oct** &
**oct2<...>2xls** scripts, but that shouldn't be really
hard. Separating the file access functions and the actual
reading/writing from/to the workbook in memory has made developer's
life (I mean: my time developing this stuff) much easier.  
  
Some
other options for development (who?):

- Speeding
  up, especially Java worksheet/cell access. For cracks, not me.

  - Automatic
    conversion of Excel date/time values into octave ones and vice
    versa. But then again Excel's dates are 01-01-1900 based (octave's
    0-0-0000) and buggy (Excel thinks 1900 is a leap year), and I
    sometimes have to use dates from before 1900. Maybe as an option?

    - Creating
      Excel graphs (a significant enterprise to write from scratch).

      - Support
        for "passing function handle" in xlsread.

Enjoy!

Philip
Nienhuis, Mar1, 2010
